# Targeting a moonlighting function of aldolase induces apoptosis in cancer cells

**DOI:** 10.1038/s41419-019-1968-4

**Published:** 2019-09-26

**Authors:** Agnieszka Gizak, Janusz Wiśniewski, Paul Heron, Piotr Mamczur, Jurgen Sygusch, Dariusz Rakus

**Affiliations:** 1 Department of Molecular Physiology and Neurobiology, University of Wroclaw, Wroclaw, 50-335 Poland; 2 Department of Biochemistry and Molecular Medicine, Université de Montréal, Montréal, Québec H3C 3J7 Canada

**Keywords:** Enzymes, Cancer therapy, Target identification, Cancer

## Abstract

Muscle fructose-1,6-bisphosphate aldolase (ALDOA) is among the most abundant glycolytic enzymes in all cancer cells. Here, we show that the enzyme plays a previously unknown and critical role in a cancer cell survival. Simultaneous inhibition of ALDOA activity and interaction with F-actin cytoskeleton using ALDOA slow-binding inhibitor UM0112176 leads to a rapid cofilin-dependent loss of F-actin stress fibers which is associated with elevated ROS production, inhibition of ATP synthesis, increase in calcium levels, caspase activation and arrested cellular proliferation. These effects can be reproduced by silencing of ALDOA. The mechanism of pharmacological action is, however, independent of the catalytic function of the enzyme, specific to cancer cells, and is most deleterious to cells undergoing the epithelial–mesenchymal transition, a process facilitating cancer cell invasion. Our results demonstrate that the overabundance of ALDOA in cancer cells is associated with its moonlighting rather than catalytic functions. This may have significant implications for development of novel broad-based anti-cancer therapies.

## Introduction

Since Otto Warburg’s finding that cancer cells exhibit extremely high rate of glucose uptake and lactate production^1^, a multitude of studies have demonstrated that the majority of cancer cells rely on glycolytic energy production^2^. This has stimulated laboratories world-wide to search for inhibitors of glycolysis as potential anticancer drugs.

However, the biological significance of the very high expression of glycolytic enzymes in cancers is enigmatic. The first enzyme of glycolysis—hexokinase, is expressed at a lower level compared to successive enzymes^3^, and has relatively low specific activity and affinity to substrates^4,5^. Thus, its total activity limits the flux through the glycolysis. Hence, the overabundance of glycolytic enzymes in cancer cells cannot promote a faster glucose catabolism but must be associated with other, noncatalytic processes.

Glycolytic enzymes belong to moonlighting enzymes^6,7^ whose physiological role is not limited to catalytic function and which can act as regulators of a variety of cellular processes. We present evidence that a glycolytic enzyme, aldolase, may be a powerful target in anti-cancer therapy and that the disruption of a complex web of intracellular interactions mediated by the enzyme is critical for its anti-cancer action.

Aldolase is positioned midway in the glycolytic pathway and catalyzes the reversible cleavage of fructose-1,6-bisphosphate (FBP) to dihydroxyacetone-3-phosphate and glyceraldehyde-3-phosphate. Its muscle isoform (ALDOA), which is the most abundant aldolase isoform in almost all cancers^8^, can organize actin filaments^9,10^, affect activities of AMPK^11,12^ and FBP2^13^, and regulate Wnt^14,15^ and p53 signaling^16^. It has been also shown that ALDOA is involved in progression of the S/G1 phase of the cell cycle^17^. Since ALDOA participates in many cellular events necessary for cancer cell survival and proliferation, we hypothesised that disruption of the moonlighting functions of ALDOA could provide a preferable target for anti-cancer therapy.

Our results reveal that in cancerous but not in normal cells, the perturbation of the ALDOA interaction with actin cytoskeleton provokes a sequence of intracellular changes, including a significant elevation of ROS production, cessation of ATP synthesis and increase in calcium levels, which result in caspase activation and blockade of cellular proliferation. Since the mechanism of these changes is independent of the catalytic function of ALDOA, we conclude that overexpression of ALDOA in cancerous cells is not related to their high glycolytic requirements but represents an adaptation by which metastatic cancer cells ensure integrity of their actin cytoskeleton while undergoing the epithelial–mesenchymal transition. Thus, the mechanism of action presented here may have significant implications for development of novel broad-based anticancer therapies.

### UM0112176—a novel ALDOA inhibitor—significantly reduces cancer cell growth

To perturb the ALDOA interaction with its binding partners, we used a slow binding mixed inhibitor of ALDOA (UM0112176; Supplementary Fig. S1a) which we found by high-throughput screening of the large compound library at the University of Montreal. UM0112176 inhibited human ALDOA activity with IC50 ∼ 2–5 μM in a slow binding manner (Supplementary Fig. S1b, c), without inhibiting other glycolytic enzymes (Supplementary Fig. S1d). We verified the ability of UM0112176 to inhibit ALDOA activity in vivo by determining cellular levels of fructose-1,6-bisphosphate and triose phosphates after 24-h incubation of cells in the presence of the inhibitor (Supplementary Fig. S1e). The results showed a significant increase in the titer of ALDOA substrate and strong decrease in ALDOA reaction products both in cancerous (KLN205, mouse lung squamous cancer) and in normal cells (primary culture of rat astrocytes). This is supported by the observed inverse trend using glutamine-only cell culture media.

**Fig. 1:**
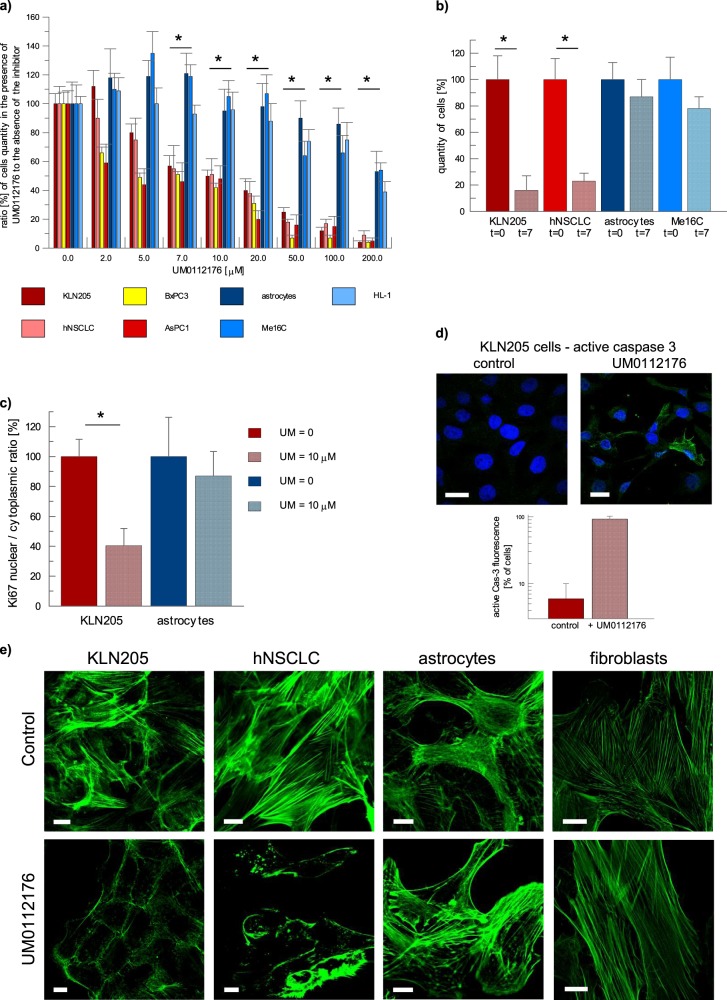
UM0112176 inhibits proliferation of cancer cells while stimulating cytoskeleton disruption and caspase activation. **a** The quantity of normal and cancer cells observed when incubated for 48 h in the presence or absence of the 10 μM UM0112176 and normalized with respect to *t* = 0. The values are given as a mean and SD, *p < 0.05 for all normal and cancer cell lines. **b** The quantity of normal and cancer cells surviving after 7-day treatment with 10 μM UM0112176. The values are given as a mean and SD, *p < 0.05. **c** The effect of 10 μM UM0112176 (24 h of treatment) on Ki67 expression in KLN205 and astrocytes. Expression of Ki67 in cells growing in the absence of the inhibitor was normalized to be 100%. The values are given as a mean and SD, *p < 0.05. **d** The activation of caspase 3 in KLN205 cells after treatment with UM0112176 (10 μM, 24 h). Bar = 20 µm. The graph below shows the number of cells [in %] exhibiting active caspase 3 fluorescence. **e** The effect of UM0112176 on the morphology of the actin cytoskeleton in cancer cell line, KLN205 and hNSCLC, and in normal cells, astrocytes and fibroblasts. Bar = 10 µm

Next, we determined a dose-response curve of UM0112176 and found that 10 µM UM0112176 strongly reduced the growth of all cancer cell lines tested: KLN205, hNSCLC (human non-small cell lung cancer cell line), BxPC3 (human pancreatic adenocarcinoma) and AsPC1 (human pancreas adenocarcinoma ascites metastasis) (Fig. 1a). The growth rate of normal cell lines (rat astrocytes, mouse cardiomyocytes—HL-1 cell line, and a human epithelial cell line—ME16C) was practically unaffected by 10 µM UM0112176 (Fig. 1a). A prolonged 7-day treatment with 10 µM UM0112176 reduced the number of cancer cells fourfold whereas normal cell count decreased only by 25–30% (Fig. 1b).

To distinguish whether the inhibition of cellular growth rate was due to elevated mortality or block in proliferation, we assayed the cellular expression of Ki67 (a marker of cellular proliferation) and active caspase (caspase 3, the final effector in apoptosis). The results revealed that in cancerous cells, 10 µM UM0112176 (24 h of treatment) strongly diminished Ki67-related fluorescent signal (Fig. 1c) and elevated the presence of active caspase (Fig. 1d). In normal cells, no changes were observed (Fig. 1d) even after 48-h treatment (Supplementary Fig. S2a).

### UM0112176 treatment disrupts actin cytoskeleton and changes ROS, mitochondrial membrane potential, Ca^2+^, DSB, and ATP levels in cancerous but not normal cells

To elucidate the mechanism of UM0112176 action we examined a number of cellular activities potentially influenced by the inhibitor, including the cytoskeleton morphology, ATP concentration, reactive oxygen species (ROS) levels, mitochondrial membrane potential and double-strand DNA breaks (DSBs). The most pronounced manifestation of the inhibitor action was a complete disappearance of F-actin stress fibres in cancer but not in normal cells after 24 h of incubation with the inhibitor (Fig. 1e). Intriguingly, the onset of the disruption of polymeric actin was much faster (<5 min) (Supplementary Fig. S3a) than full inhibition of ALDOA in vitro (∼15 min). This supports the hypothesis that F-actin depolymerization is not dependent on the inhibition of ALDOA activity.

**Fig. 2:**
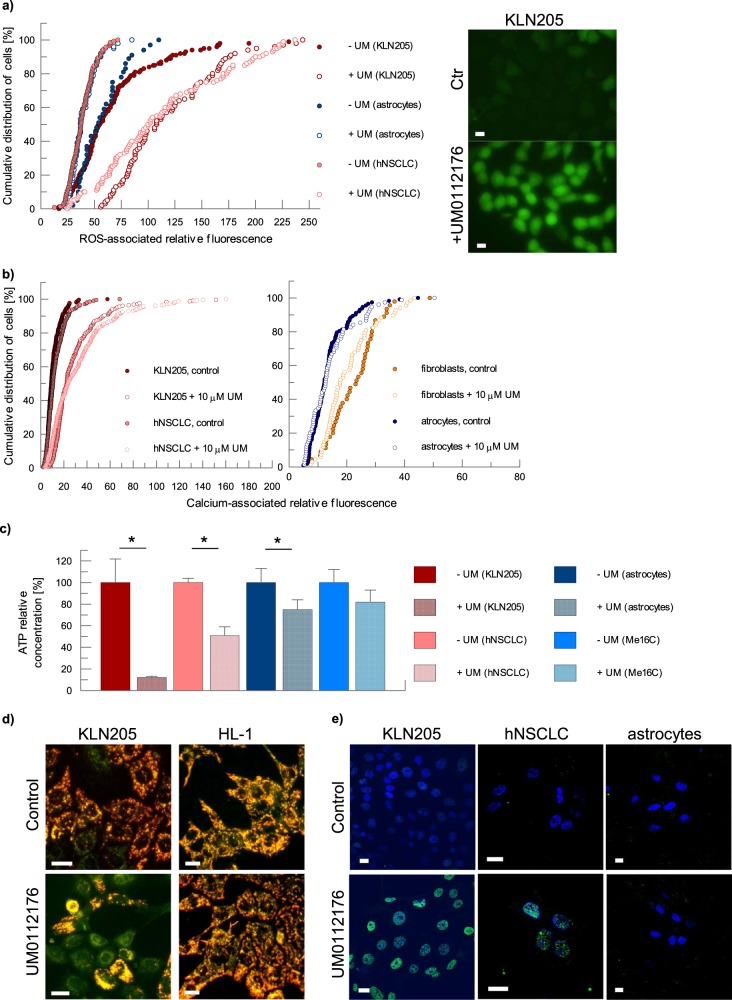
The pleiotropic effect of UM0112176 on cellular physiology. **a** The effect of UM0112176 incubation (8 h) on ROS levels in KLN205 and NSCLC cells as well as in astrocytes; images (right) show ROS-associated fluorescent signal in KLN205 cells. Bar = 20 µm. **b** The influence of the inhibitor on Ca^2+^ concentration in cancer and normal cells. **c** The reduction of cellular ATP levels after UM112176 treatment in cancer and normal cells. The values are given as a mean from 3 measurement for 3 independent experiments and SD, *p < 0.05. **d** Polarization of mitochondrial membrane in cancer and normal cells upon UM0112176 treatment. The more yellow appear the mitochondria the higher is the ratio of JC-1 aggregates (red, strong polarization) to monomers of the dye (green, depolarized). Bar = 10 µM. **e** The induction of DSB (green) by UM112176 treatment (8 h) in KLN205 and hNSCLC cells. Nuclei were counterstained with DAPI (blue). Bar = 10 µm

Along with the disruption of cytoskeleton, we observed a significant elevation in ROS and calcium levels, a decrease in ATP concentration and in the mitochondrial membrane potential, as well as the induction of double-strand DNA breaks (Fig. 2a–e). Importantly, UM0112176-induced changes in cancer cells were not transmitted (e.g., by ROS release) to normal cells: UM0112176-treatment of KLN205 cells co-cultured with normal human fibroblasts elevated ROS levels only in cancer cells (Supplementary Fig. S3b).

### UM0112176-induced changes cannot be explained through inhibition of glycolysis

To further address whether UM0112176-induced changes can be reproduced by inhibition of glycolysis in cancer cells, we tested the impact of other glycolytic enzymes effectors: alizarin Red S which inhibits phosphoglycerate mutase (PGAM)^18^ and 3-PO ((2E)-3-(3-pyridinyl)-1-(4-pyridinyl)-2-propen-1-one) which indirectly blocks the action of 6-phosphofructo-2-kinase/fructose-bisphosphatase-2 (PFK-2)^19^. Inhibition of PGAM resulted in an increase in ROS (Supplementary Fig. S4a) having no effect on cytoplasmic calcium level while inhibition of PFK-2 elevated cytoplasmic Ca^2+^ levels without changes in ROS (Supplementary Fig. S4a, b). Furthermore, even rapidly proliferating cancer cells (KLN205), were able to maintain a constant level of total ATP (Supplementary Fig. S4c, d) in presence of these inhibitors presumably using other nutrients such as glutamine rather than activating glucose uptake. Importantly, neither 3-PO nor Alizarin Red S disrupted the F-actin cytoskeleton or induced DSBs (data not shown).

### UM0112176 disrupts ALDOA–actin interactions

Aldolase is known to interact with actin and actin-binding proteins^8,9^, influencing cytoskeleton dynamics. It can inhibit WASP-dependent actin polymerization^14^ and lead to cytokinesis defect^20^ but can also promote actin-dependent processes such as cancer cell motility acquired in the EMT transition^21^. This complex role of ALDOA evokes the hypothesis that UM0112176 binding to ALDOA may be responsible for the observed disruption of the F-actin cytoskeleton.

**Fig. 3:**
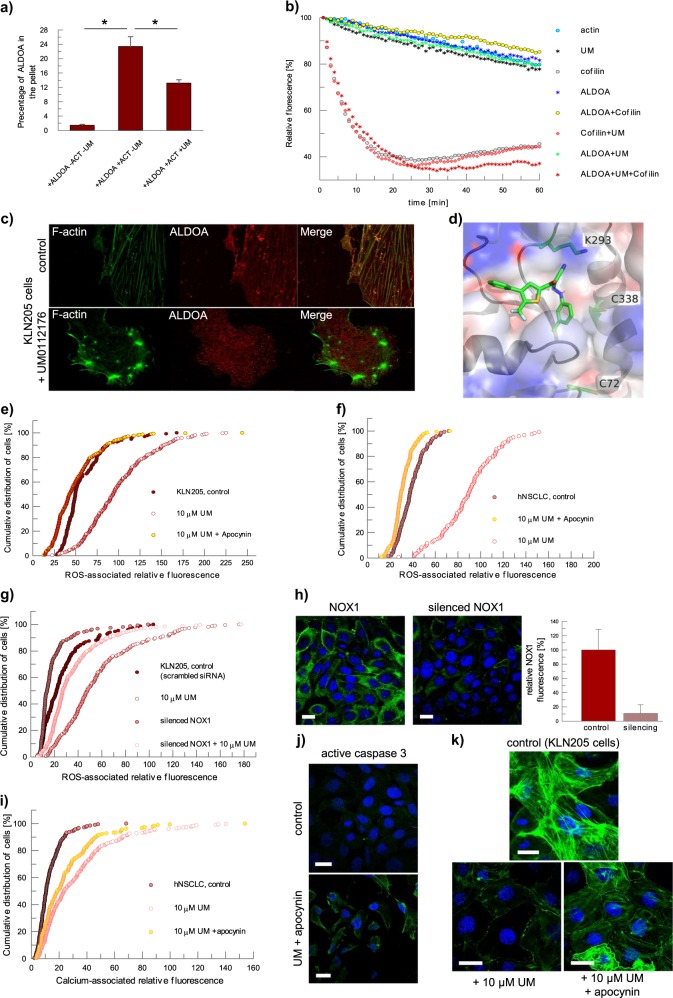
The disruption of the ALDOA–actin filament interaction stimulates NOX1-mediated elevation of ROS and Ca^2+^ levels in cancer cells. **a** The UM0112176-induced partial dissociation of ALDOA from β/γ actin filaments in vitro. The values are given as a mean and SD, *p < 0.05. **b** The impact of various combinations of UM0112176, ALDOA and cofilin on the depolymerization of β/γ actin. **c** UM0112176-induced ALDOA dissociation causes F-actin bundling and depolymerization in KLN205 cells. **d** Docking result of UM0112176 to ALDOA using Autodock. Inhibitor UM0112176, and residues C72, K293, and C338 are shown in stick style, while the ALDOA fold is shown in cartoon style. All oxygen atoms are colored in red, carbon in green, sulfur atoms in yellow, and nitrogen atoms are colored in blue. Charges on the electrostatic potential surface of ALDOA are represented in blue for regions of positive charge and red for regions of negative charge. Figure elaborated with PyMol (The PyMOL Molecular Graphics System, Version 2.0 Schrödinger, LLC.) **e**, **f** Apocynin diminishes UM0112176-induced ROS levels in KLN205 and hNSCLC cells, respectively. **g** NOX1 silencing diminishes ROS levels in KLN205 cells treated with UM0112176. **h** NOX1-associated fluorescent signal (green) before and after its silencing in KLN205 cells. Nuclei were counterstained with DAPI (blue). Bar = 20 µm. The graph on right shows a decrease in NOX1-associated fluorescence after silencing. **i** Apocynin partially protects rise in calcium levels after UM0112716 treatment. **j** The effect of apocynin on caspase 3 activation. The image shows the activated caspase 3 (green) in KLN205 cells. Bar = 20 µm. **k** Apocynin prevents disruption of the actin cytoskeleton in cells treated with UM0112176. Bar = 20 µm

The cytoskeleton disruption is, however, actin isoform-specific as UM0112176 partially disrupted the association of ALDOA with the fibrillar form of cytoskeletal actin containing β and γ isoforms (Fig. 3a) while having no effect on the stability of the muscle (α isomer) actin polymers (data not shown). In F-actin-aldolase rafts, F-actin contacts with aldolase are found primarily in two loci; the region of residues 1–8 and residues 350–365^22^. Antigenic probes located tight binding sites for aldolase in conserved C-terminal regions of α-actin microfilaments^23^. A Brownian dynamics simulation found similar actin residues that participate in aldolase binding^24^. As differences in residues 1–8 distinguish the actin isoforms^25^, the actin N-terminal region thus drives isoform-specific binding with aldolase. ALDOA has also been shown to preferentially interact with the γ actin in cellular pulldowns of lung cancer cell lysates^26^ corroborating that ALDOA contact with the actin N-terminal region distinguishes between the actin isoforms.

Interestingly, the ALDOA binding site overlaps with the binding site of cofilin on actin^27,28^, a protein known to accelerate depolymerization of actin fibres^29^, whose binding implicates overlapping N- and C-terminal actin residues^30^. Given the large globular shapes of ALDOA and cofilin, the extensive apparent overlap in actin-binding sites indicates competition by ALDOA and cofilin for the same binding loci on actin. The incubation of F-actin (β-γ isoforms) with both ALDOA and cofilin (in equimolar subunit concentrations) showed little effect of cofilin on F-actin stability (Fig. 3b) indicating that ALDOA protected F-actin against cofilin action. This is consistent with the tighter expected binding of ALDOA to F-actin^23^ than cofilin which has an estimated dissociation constant of ∼10 μM^31^. The in vitro protection by ALDOA against the cofilin-mediated depolymerization of actin was abolished with the addition of UM0112176 (Fig. 3b). Although ALDOA could interact with cofilin in vitro, we found no evidence for UM0112176 to perturb this interaction (Supplementary Fig. S2b). The destabilization of the F-actin cytoskeleton by UM0112176 must thus arise from dissociation of ALDOA from its binding loci on F-actin that overlaps with the cofilin binding loci, thereby enabling cofilin attachment to F-actin and mediating its depolymerization. In line with this, we observed the absence of colocalization of cytoskeletal actin and ALDOA in KLN205 cells treated with UM0112176 (Fig. 3c).

To investigate the interaction of UM0112176 with ALDOA (PDB: 1ZAJ) a blind molecular docking analysis was performed. Calculations using AutoDock^32^ indicated 1 major and 2 minor pose clusters for UM0112176 on ALDOA. The major cluster contained 17 poses with predicted binding energy near ∼9.2 kcal/mole while the minor cluster had each 3 poses of slightly lower binding energy. Visual inspection places the top pose vicinal to Lys-293 and Cys-338 (Fig. 3d) enabling their rotamers to hydrogen bond, respectively with −C≡N and –Cl functional groups of UM0112176. Modification of Cys-72 and Cys-338 either by their crosslinking^33^ or by oxidized glutathione inhibits catalytic activity^34^ through long range communication with the active site ~25Å distant. Long range inhibition of dynamical events during catalysis requisite for ALDOA activity by UM0112176, as was shown for oxidized glutathione inactivation of ALDOA^34^, is consistent with its inhibition kinetics. Intriguingly, Lys-293 has been shown to promote γ-actin interaction with ALDOA as K293A mutation abrogates the binding^26^. Furthermore, the residues comprising the UM0112176 binding cavity overlap with those shown for raltegravir on ALDOA and treatment with raltegravir (100 μM) prolonged the mouse survival rate ∼2-fold relative to the control group^26^ in an orthotopic lung cancer model. We hypothesise that UM0112176 leverages the ability to dissociate ALDOA from β/γ actin by competing with γ-actin for the interaction with Lys-293 on ALDOA.

This finding does not, however, explain the absence of UM0112176 action on cytoskeletal actin in normal cells. Evidently, in normal cells, other actin-associated proteins must protect the cytoskeleton against the cofilin-induced depolymerization but in cancer cells, this protection is apparently defective. We, therefore, searched for cancer-specific factors which could accelerate depolymerization of the F-actin cytoskeleton and would be capable of inducing apoptosis. We found that one of the prominent cellular effects of UM0112176 application was a very rapid intensification of ROS production which occurred exclusively in cancer cells (Fig. 2a). As the increase in ROS levels was concomitant with the modulation of the F-actin fiber structure^35^, we focused on cytoskeleton-associated sources of ROS which may be specific for cancer cells.

### Increase of ROS levels after UM0112176 treatment is caused primarily by NOX activity

NADPH-dependent oxidase (NOX) is a significant source of intracellular ROS that can be regulated by the actin cytoskeleton^36^. It has been demonstrated that NOX1 participates in epithelial–mesenchymal transition, a process facilitating cancer cell invasion^37^. Thus, we hypothesised that UM0112176-induced displacement of ALDOA from polymerized actin enabled cofilin-directed destabilization of the cytoskeleton and that ensuing depolymerization may be responsible for the ROS increase. Intriguingly, in most cancer cells, NOX proteins (especially NOX1 isoform) are expressed at a higher level than in normal cells^38^ (Supplementary Fig. S2c). Thus, we silenced NOX1 expression in cancer cells (Fig. 3h) or inhibited NOX1 activity using apocynin^39^. The resultant reduction in NOX1 activity and expression blocked the UM0112176-induced elevation of ROS levels (Fig. 3e–g).

This actin-dependent mechanism of ROS production suggests that UM0112176 by stimulating displacement of ALDOA from actin filaments enables cofilin to depolymerize actin filaments and thus, activate NOX. ROS production by activated NOX, in turn, enables redox activation of slingshot homolog 1 to dephosphorylate P-cofilin^40^. Indeed, the addition of UM0112176 reduced cellular P-cofilin levels (Supplementary Fig. S5a). ALDOA dissociated from actin filaments by UM0112176 action promotes their further depolymerization by releasing additional binding sites for dephosphorylated cofilin to bind with the filaments. This feed-forward mechanism would explain the rapid depolymerization of actin observed in KLN205 and hNSCLC cells.

Surprisingly, apocynin only partially halted the UM0112176-induced increase in cytoplasmic calcium level and it did not protect against caspase 3 activation (Fig. 3i, j). Although apocynin apparently inhibited disruption of actin filaments by UM0112176, the cytoskeleton appeared more bundled compared to untreated cells, and cells displayed an altered morphology when they were simultaneously treated with apocynin and UM0112176 (Fig. 3k). The perturbation of the cytoskeleton by either UM0112176 or in combination with apocynin is consistent with NOX1-dependent ROS production for proper cytoskeletal organization in these cancer cells.

### Cellular mechanism of UM0112176 action involves mitoK_ATP_ opening

A protein that could potentially link the actin cytoskeleton with mitochondrial ROS production and apoptosis is the mitochondrial ATP-dependent potassium channel (mitoK_ATP_). A link between intracellular K^+^ concentration and apoptosis has been documented in many systems and has implicated mitoK_ATP_^41^. The channel appears to be sensitive to depolymerization of actin that increases its opening^42,43^ and results in mild uncoupling and increase in mitochondrial ROS production^44^. There is, however, controversy in the literature, with some results suggesting that rather actin disruption closes mitoK_ATP_^45^.

**Fig. 4:**
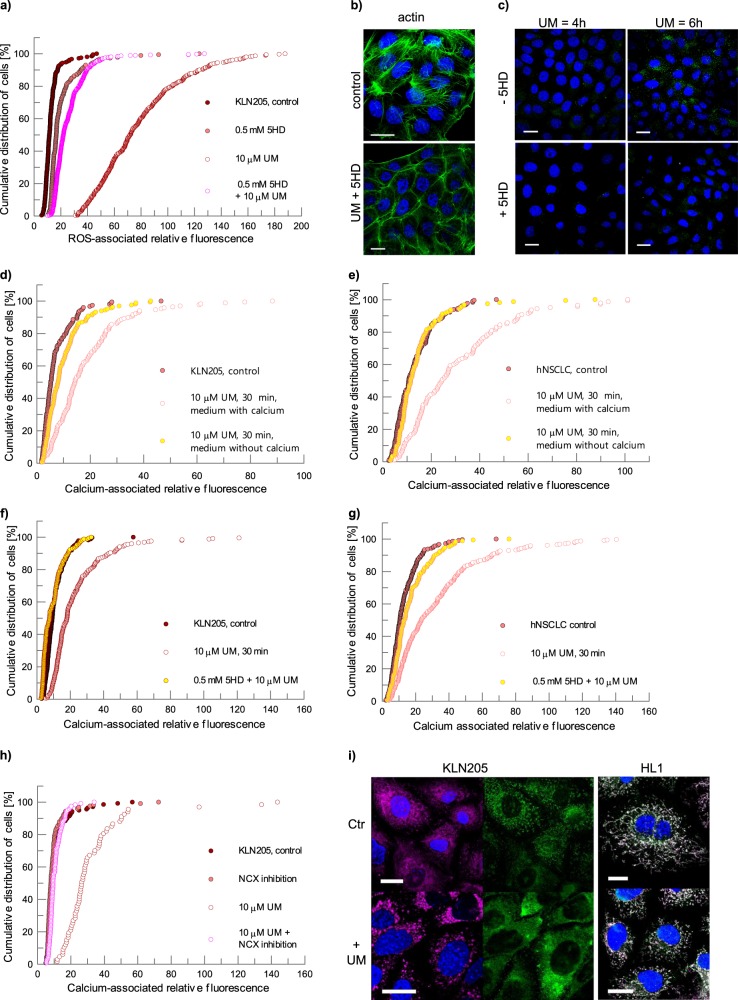
Mechanism of UM0112176-induced changes in cancer cells. **a** 5HD inhibits ROS levels in UM0112176 treated KLN205 cells. **b** 5HD does not protect against UM0112176-induced actin cytoskeleton (green) disruption. **c** Diminished caspase 3 (green) activation in cells pretreated with 5HD before UM0112176 incubation. **d**, **e** Ca^2+^ influx from external sources after UM0112176 stimulation of KLN205 and hNSCLC cells, respectively. **f**, **g** The effect of mitoK_ATP_ channel inhibition on the UM0112176-induced [Ca^2+^] increase in KLN205 and hNSCLC cells, respectively. **h** The reduction of UM0112176-induced calcium influx after inhibition of NCX in KLN205 cells. **i** The effect of UM0112176 on HK2 (green) interactions with mitochondria (magenta) in KLN205 cancer cells and normal HL-1 cells. To better visualize changes in HK2 localization in KLN205 cells (the left side of panel **i**), the green channel (HK2) is presented separately from merged magenta (mitochondria) and blue (nuclei) channels. For HL-1 cells, white color indicates localization of green-stained HK2 and magenta-colored mitochondria. Bar = 20 µm

We pre-treated cancer cells with 5HD (5-Hydroxydecanoate), which is known to block opening of mitoK_ATP_^44^, and we observed that 5HD considerably diminished the UM0112176-induced increase in ROS (Fig. 4a). 5HD pre-treatment was not able to block the actin cytoskeleton disruption (Fig. 4b) and induction of apoptosis by UM0112176, although it significantly slowed down the rate of caspase 3 activation (Fig. 4c). Thus, UM0112176-driven depolymerization of cytoskeletal actin may be responsible for ROS production both by opening of the mitoK_ATP_ and, independently, by NOX1 activation which in turn, potentiates the cytoskeleton disruption.

### The reverse mode of the sodium-calcium exchanger is responsible for UM0112176-induced calcium influx

UM0112176 binding to ALDOA was also associated with a significant increase in cellular calcium level in cancer but not in normal cells (Fig. 2b). This increase was more rapid in KLN205 (<5 min) than in hNSCLC cells (~20 min) (Fig. S4g). We, therefore, searched for the origin of this calcium and found that it originated from the extracellular store (Fig. 4d, e). Depletion of Ca^2+^ from culture medium prevented the UM0112176-induced increase of cellular calcium.

We then asked whether NOX1 and mitoK_ATP_ might synergistically regulate the changes of the intracellular free Ca^2+^ level. Indeed, inhibition of NOX1 partially protected against UM0112176-induced Ca^2+^ increase (Fig. 3i), while inhibition of mitoK_ATP_ opening virtually abolished the increase (Fig. 4f, g). Since neither protein functions as a calcium channel or exchanger, they must function as regulators (e.g., via ROS) of proteins directly involved in calcium homeostasis.

The activation of NOX can lead to mitoK_ATP_ opening which, in turn, can indirectly stimulate the reverse mode of the sodium-calcium exchanger (NCXrev), promoting the influx of Ca^2+^ into cells^46^. Thus, we tested a possible involvement of NCX in the UM0112176-induced increase in calcium. Indeed, inhibition of the NCXrev by KB-R7943^46^ reduced the calcium influx (Fig. 4h). However, this did not prevent the induction of apoptosis (Supplementary Fig. S5b).

It has been shown that actin depolymerization stimulates the NCXrev activity^47^ which lends further support for NCX as the exchanger responsible for Ca^2+^ influx upon UM0112176 addition.

Therefore, it can be concluded that the crosstalk between NOX and mitochondrial ROS^48^ activates membrane transporters, sodium/hydrogen exchanger and sodium/bicarbonate cotransporter via the stimulation of the ROS-sensitive MAPK cascade and culminates in Ca^2+^ influx via NCXrev^49^. Furthermore, cytoplasmic Ca^2+^ overload activates NOX^50^ and mitochondrial Ca^2+^ loading via the mitochondrial calcium uniporter accelerates mitochondrial ROS production^51^, thereby creating a positive feed-back loop that maintains high ROS and calcium levels and induces apoptosis. The suppression of the Ca^2+^ influx by inhibition of the NCXrev (Fig. 4h) supports the notion that the opening of mitoK_ATP_ leading to mitoROS release into cytoplasm^52^ constitutes the crucial event responsible for apoptosis.

### Cancer cell-specific decrease in ATP levels upon ALDOA inhibition induces a rapid dissociation of HK2 from mitochondria

Although UM0112176 inhibited ATP synthesis in all cell lines, ATP changes were larger and more rapid in cancers cells (Fig. 2c). Importantly, in normal but not cancer cells, these changes were reversible upon withdrawal of the inhibitor (Supplementary Fig. S4e). The addition of UM0112176 induced a rapid decline (<5 min) in ATP levels in KLN205 (∼40% decline) and hNSCLC (∼20% decline) cells (Supplementary Fig. S4f) while no such changes were observed in normal cells (Supplementary Fig. S4f).

This biphasic kinetics of UM0112176-induced ATP depletion raised the question of the mechanism responsible for the fast decrease of ATP levels in cancer cells. Therefore, we examined the subcellular localization of hexokinase 2 (HK2) whose expression is elevated in most cancer cells and which requires association with mitochondria for full activity and efficient ATP synthesis by mitochondria^4,5^. We observed that in cancer but not in normal cells, HK2 dissociated rapidly from mitochondria after UM0112176 treatment (Fig. 4i) and the timespan of these changes was correlated with the timescale of ROS production. Thus, in rapidly proliferating cancer cells, the first phase of the UM0112176-dependent decrease in ATP levels correlates with the rapid dissociation of HK2 from mitochondria.

The silencing of ALDOA expression (Supplementary Fig. S5c) recapitulated the observations induced by UM0112176 treatment (Supplementary Fig. S5d–f) and showed no concomitant decrease of ATP levels, in agreement with results reported by Lew and Tolan^14,20^. This argues that the observed decrease of ATP levels is not a consequence of a lower metabolic activity but originates from moonlighting interactions of ALDOA with its binding partners that are abrogated by UM0112176. Chang et al. pointed out that the ALDOA mutation of K293A influences the ALDOA interaction with γ-actin but does not entail changes in glycolytic flux^26^ supporting our interpretation that UM0112176 interferes with the moonlighting interactions of ALDOA.

### The effect of UM0112176 on cancer cells cannot be explained by FBP accumulation

To verify if the observed changes are associated with non-metabolic function of ALDOA or if they were consequences of the inhibition of enzyme activity and hence, accumulation of FBP, we examined the effect of FBP on ROS and ATP level as well as on the cytoskeleton structure. Although the efficiency of transmembrane diffusion of FBP is relatively low, it may be actively and effectively transported by variety of cells^53,54^. We observed that 20 mM but not 5 mM extracellular FBP stimulated ROS production and destabilized cytoskeleton structure in cancer cells (Supplementary Fig. S3c, d) which is consistent with the long range communication between the aldolase active site and UM0112176 binding locus. The same FBP concentrations had only a minor effect on normal cells (Supplementary Fig. S3c, d), mirroring the trend observed for cellular treatment with UM0112176. The lack of discernable changes in astrocytes agrees with the virtual absence of NOX1 in these cells (Supplementary Fig. S2c) and reinforces the importance of ROS production by NOX1 in cancer cells for proper cytoskeletal organization. Although the presence of low levels of NOX1 in normal cells that do not undergo apoptosis when treated with UM0112176 could argue against NOX1 conferring cancer cell selectivity during UM0112176 treatment, it was shown that low glucose culture media (5 mM) prevented the NOX4 isoform from targeting to lipid rafts in adipocytes retaining it in a low activity membrane fraction^55^. As our cell culture experiments were conducted using concentrations of glucose which model the physiological environment (5 mM), the presence of NOX1 in normal cells likely serves some other non-cytoskeletal function.

### UM0112176 prevents nuclear localization of ALDOA

Unexpectedly, UM0112176 treatment also resulted in the depletion of ALDOA from nuclei of cancer cells (Supplementary Fig. S5g). Nuclear ALDOA localization has been shown to be associated with cell cycle progression^17^, however, the mechanism of ALDOA nuclear transport and its significance for regulation of the cell cycle requires further studies.

## Conclusions

**Fig. 5:**
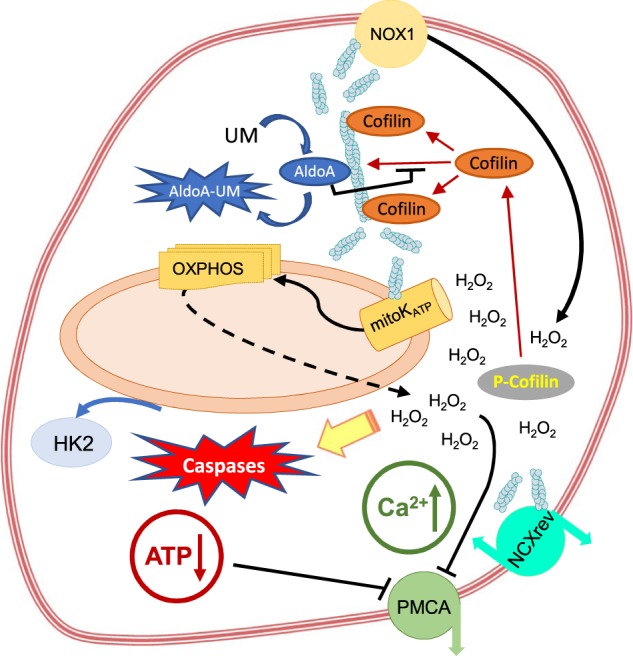
Scheme of UM0112176-evoked changes in cancer cells. UM0112176 binding to ALDOA disrupts its interaction with fibrillar actin enabling cofilin binding which initiates depolymerization of the actin cytoskeleton. This, in turn, activates ROS production by NOX1 and further stimulates cofilin dephosphorylation that accelerates cytoskeleton disruption. ROS levels stimulate mitoK_ATP_ function, decrease in mitochondrial membrane potential (ΔΨ↓) and this effect is enhanced by F-actin depolymerization. Elevated levels of ROS, generated by mitochondria and NOX1, stimulates the reverse mode of NCX action resulting in an increase in intracellular levels of Ca^2+^. ROS-dependent dissociation of HK2 from mitochondria decreases ATP level and together with increasing ROS levels results in the inhibition of PMCA (plasma membrane Ca^2+^ ATPase) preventing Ca^2+^ efflux. The elevated ROS levels in the cytoplasm initiates caspase activation

Here, we have presented evidence that perturbation of the ALDOA interaction with F-actin by a low molecular weight aldolase inhibitor promotes a series of cellular events leading to inhibition of cancer cell proliferation and to stimulation of apoptotic cell death as outlined in Fig. 5. The overexpression of ALDOA in cancer cells represents a mechanism by which metastatic cancer cells ensure integrity of their actin cytoskeleton while undergoing the epithelial–mesenchymal transition. The pharmacological mechanism of action discovered during our studies may have significant implications for development of novel pan-anticancer therapies.

## Methods

### Experimental model

All the cell culture media used in the study were from Sigma. All cell lines and primary cell cultures were maintained at 37 °C under 5% CO_2_. If not stated otherwise, prior to the experiments the cells were cultured for 48 h under standard conditions.

### Cell lines

HL-1 cells (mouse female; grown in Claycomb medium) were provided by Dr. W.C. Claycomb Louisiana State University Health Science Center, New Orleans, LA, USA), who first established and characterized the cell line. KLN-205 (mouse; grown in DMEM), AsPC1 (human female; grown in RPMI 1640), BxPC3 (human female; grown in RPMI 1640), Me16C (human female; grown in MEMB) cells were purchased from ATCC.

### Primary cell cultures

Normal human dermal fibroblasts (grown in DMEM) were from Lonza. Mouse astrocytes (female; grown in DMEM with D-valine to prevent fibroblasts outgrowth) were isolated and cultured as described before^56^. To prepare an explant-derived primary culture of human non-small lung cancer cells, histologically proven small cell lung tumor fragments were cut into ∼1 mm^3^ sections and placed into culture dishes coated with Matrigel (hNSCLC; female; grown in DMEM with D-valine to prevent fibroblasts outgrowth). To verify the cellular purity of explant-derived hNSCLC cell line, immunostaining was performed to detect cytokeratin-7, a cancer cell marker (data not shown).

### Cell treatment with inhibitors and other compounds

The chemicals listed below (except UM0112176) were bought from Sigma. The following concentrations of inhibitors and other compounds were used in this study: soluble in DMSO: 1–200 µM UM0112176, 10 µM apocynin, 5 µM 3PO, 5 µM KB-R7943; soluble in water: 10 µM alizarin red S, 5- and 20-mM fructose-1,6-bisphosphate; 0.5 mM 5-hydroxydecanoid acid. In the experiments, the final concentration of DMSO in culture media did not exceed 0.2%.

### Immunocytochemistry

Cells growing on coverslips were fixed in 4% paraformaldehyde, permeabilized with 0.1% Triton X-100 in PBS and incubated with 3% BSA in PBS to reduce unspecific binding of antibodies. Then cells were incubated overnight with a primary antibody (mouse anti-ALDOA, 1:200^57^; rabbit anti-HK2, 1:300, Sigma; rabbit anti-Ki67, 1:100, Novocastra; mouse anti-γ-actin, 1:500, Sigma; rabbit anti-β-actin, 1:500, Sigma; rabbit anti-caspase 3, 1:800, Sigma; rabbit anti-H2AX gamma, 1:500, GeneTex) and 1 h with an appropriate secondary antibody (anti-rabbit Alexa 633, 5 µg/mL, ThermoFisher Scientific; anti-rabbit FITC, 1:600, Sigma; anti-mouse TRITC, 1:300, Sigma). To visualize nuclei, actin cytoskeleton, and mitochondria, the cells were counterstained with respectively, DAPI (Sigma), Phalloidin-Alexa 488 and MitoTracker Deep Red FM (ThermoFisher). Images were acquired on FV-1000 confocal microscope (Olympus) with ×60 (oil, Plan SApo, NA = 1.35) objective. The fluorochromes were excited at 405 (DAPI), 473 (Alexa 488), 559 (TRITC) and 635 (Alexa 633 and MitoTracker Deep Red FM) nm and imaged using the Sequential scan option. The quantification of nuclear/cytoplasmic ALDOA fluorescence signal ratio was performed using the Cell^F software (Olympus). The nucleus and cytoplasm of the same cell were marked and the mean fluorescence within the marked areas was measured. The measurements were taken from at least 70 cells (usually over 100 cells) from randomly selected areas. All cells observed in the areas were used for measurements. The corrected total cell fluorescence (CTCF) of individual cells was calculated using the Cell^F. For the evaluation of statistical significance a Student’s *t*-test was used. A probability of *P* < 0.05 was considered to represent a significant difference. The results were expressed as a mean and standard deviation.

### Measurement of cellular ROS and Ca^2+^ content

To measure intracellular ROS production the cells growing on coverslips were loaded with 5 μM dihydrofluorescein diacetate (H2DCF-AC, Sigma; 20 min, 37 °C), thoroughly rinsed with Hank’s Balanced Salt Solution, and mounted on slides. Live cells were examined with the Olympus IX71 fluorescence microscope equipped with the Cell^F software (Olympus). The fluorescence of the dye was excited at 488 nm for 500 ms^58^.

The Ca^2+^-sensitive fluorescent dye Fluo-3-AM was used to detect the relative change of the Ca^2+^-dependent fluorescence in cells. Cells were loaded for 30 min with 5 µM dye in amino-acids- and serum-free medium, washed twice and incubated for 15 min in full culture medium (de-esterification of the dye). The fluorescence was measured with the FV-1000 confocal microscope (Olympus) using excitation and emission wavelengths of 488 and 525 nm, respectively. The measurements were taken from at least 70 cells (usually over 100 cells) from randomly selected areas. All cells observed in the areas were used for measurements. The corrected total cell fluorescence (CTCF) of individual cells was calculated using the Cell^F software (Olympus) and presented in percentage frequency graphs (“cumulative distribution”) graphs. The experiments were performed in triplicate, with similar results.

### Mitochondrial membrane potential

Loss of the mitochondrial membrane potential after 8 h incubation with 10 μM UM0112176 was detected using the fluorescent dye, JC-1 (Mitochondrial Permeability Transition Detection Kit, AbD Serotec), according to the manufacturer’s instructions. Polarized mitochondria accumulate more of the dye and become red. In cells with depolarized mitochondria, most dye is dispersed in the cytoplasm and has green fluorescence. The ratio of red to green fluorescence reflects the degree of the mitochondrial membrane polarization. Excitation of the dye was at 488 nm and the emission was observed using a long pass filter, which allowed for simultaneous observation of green (monomers of JC1) and red (aggregates of the dye) fluorescence.

### NOX1 and ALDOA expression silencing

Small interfering RNA (siRNA) against NOX1 and ALDOA and control siRNA were purchased from Sigma and Santa Cruz Biotechnology Inc., respectively, and the procedure of NOX1 and ALDOA expression silencing was performed according to the manufacturer’s instruction, using Lipofectamine 2000 (Invitrogen) as the transfection reagent.

### MTT assay

Cells growth/viability was tested using MTT assay as described previously^58^. Briefly, the cells were seeded into 96-well plates and cultured for an appropriate time with or without the tested compounds before the MTT assay. The absorbance was measured at 570 nm with a reference wavelength of 670 nm using a plate reader ASYS UVM340 (Biogenet). The amount of respective viable cells obtained after 24 h culture in control conditions was assumed to be 1.

In all experimental conditions, measurements were averaged from at least eight wells. Statistical significance of differences in the means of control and experiment groups was tested using the T test at significance level of 0.05. Dispersion was described using standard deviation. The experiments were performed in triplicate, with similar results.

### ATP measurements

ATP level in cells’ extracts was measured by the firefly bioluminescence assay using luciferin/luciferase method according to the procedure described by the supplier (Firefly Lantern Extract, Sigma). ATP was extracted by boiling (99 °C, 45 s) of cells in a buffer: 50 mM Tris, 1 mM EDTA, 0.1% Triton X-100. pH 7.4, *t* = 25 °C). Samples were centrifuged at 10,000 × *g*, 10 min, 4 °C and subjected to measurement using Turner Designs TD 20/20 luminometer.

### Fructose-1,6-bisphosphate and triose phosphates concentration in astrocytes and KLN205 cells

The cells were cultured as described in the “Experimental model” section, in the presence of 5 mM glucose and 2 mM glutamine or in the presence of only one of the energetic substrates to 50% confluence. Then the cells were incubated for 8 h with 10 µM UM0112176. Next, the cells were harvested and after washing with PBS, they were suspended in 0.3 mL of PBS and frozen in liquid nitrogen. After thawing, cells were sonicated, and protein concentration was determined using the Bradford method. The obtained samples were deproteinized by 0.3 mL of 8% cold HClO_4_, centrifuged and the supernatants were neutralized by a 2 M KOH. The obtained aliquots were used for FBP and triose phosphates enzymatic determination.

FBP was measured in a mixture (0.5 mL) containing: 50 mM TRIS, 5 mM MgCl_2_, 100 mM KCl, 0.1 mM EDTA, 0.2 mM NADH, 5 U triose-phosphate isomerase (Sigma), 2 U glyceraldehyde 3-phosphate dehydrogenase (Sigma) and 50 µL of cellular extract, pH 7.4, t = 37 °C. The reaction was initiated by 5 U aldolase (Sigma).

The concentration of triose phosphates (a sum of dihydroxyacetone phosphate and D-glyceraldehyde 3-phosphate) was assayed in: 50 mM TRIS, 5 mM MgCl_2_, 100 mM KCl, 0.1 mM EDTA, 0.2 mM NADH, 5 U triose-phosphate isomerase (Sigma) and 50 µL of cellular extract, pH 7.4, *t* = 37 °C, V = 0.5 mL. The reaction was started with 2 U glyceraldehyde 3-phosphate dehydrogenase (Sigma).

All measurements were repeated three times using cells extracts prepared from three independent cell cultures. Statistical significance of differences in the means of control and experiment groups was tested using the T test at significance level of 0.05. Dispersion was described using standard deviation.

### Protein expression and purification

Human aldolase A was expressed in *E. coli* and purified according to the following protocol. Clonal colonies of Hi-control BL21(DE3) cells (Lucigen) carrying pETite vectors with inserts encoding tag-free Aldolase A were grown on agar-LB (A&A Biotechnology) plates with 30 μg/mL kanamycin (Sigma). Randomly selected clones were used to inoculate 3 mL LB preculture and incubated overnight in a shaker incubator set to 37 °C, 200 RPM. 500 mL of LB was inoculated with 2 mL of the pre-culture and grown in 37 °C, 180 RPM for 4 h. Expression of Aldolase A was induced by addition of IPTG (A&A Biotechnology) to a final concentration of 100 μg/mL. Proteins were expressed for 6 h. Cells were pelleted by centrifugation at 4000 × *g*, 10 min, 4 °C and lysed using BugBuster (MERCK). Cellular debris was removed by centrifugation at 17,000 × *g*, 25 min, 4 °C. Two steps of out-salting were performed using 35 and 65% saturation of ammonium sulfate. Aldolase A-containing precipitant was dissolved in 25 mM TRIS-HCl buffer, pH 7.5 and dialyzed against same buffer. The protein solution was then incubated with cellulose phosphate (Sigma) at pH 7.1 for 1 h and loaded on 10 mL Pierce Centrifuge Columns (ThermoFisher Scientific). The column bed was repeatedly washed with 25 mM TRIS-HCl buffer pH 7.0, 1500 × *g*, until no absorption peak at 280 nm wavelength could be seen in spectrum of the flow through. Aldolase A was eluted with 2 M KCl. Purity of the isolated protein was checked using SDS-PAGE and the activity of aldolase was monitored as described in the “Kinetics of aldolase A in the presence of UM0112176” section at 37 °C using an HP 8453 diode array spectrophotometer.

Rabbit muscle aldolase A which is essentially identical to the human isoform (98% sequence identity; 100% sequence homology) was purified as previously reported^10^. Briefly, plasmid pPB14 coding for rabbit muscle aldolase^59^ was transformed and overexpressed in Escherichia coli strain BL21-SI (Invitrogen). Recombinant rabbit muscle aldolase was purified by a combination of anion and cation exchange chromatography and size exclusion chromatography. The activity of aldolase was monitored as described in the “Kinetics of aldolase A in the presence of UM0112176” section at 37 °C using an HP 8453 diode array spectrophotometer.

### Kinetics of aldolase A in the presence of UM0112176

Rabbit muscle aldolase activity was measured spectrophotometrically by monitoring the decrease in NADH concentration at 340 nm as described originally by Racker in 1947^60^. The assay mixture consisted of 50 mM Tris-Acetate, pH 7.5, 0.3 mM NADH, and coupling enzymes (5 μg/mL GDH and 0.5 μg/mL TIM). UM0112176, previously reconstituted in 100% DMSO to 20 mM, was then added to the assay mixture containing different concentrations of substrate FBP (5–1000 μM) at each UM0112176 concentration (25, 50, and 100 μM). DMSO concentration was controlled across assays to a final concentration of ≤0.4% DMSO. Enzyme activity was tested up to a concentration of 5% DMSO with no reduction in activity (data not shown). Enzymatic inhibition was determined from measuring residual activity upon the addition of aldolase (0.15 μg). Percentage errors in kinetic measurements were assessed to be 15% at low residual activities and less at higher activities. Controls contained assay buffers without UM01122176 and did not impact the kinetic parameters Vm and Km of the aldolase cleavage reaction. The inhibition kinetics was analyzed using the enzyme models for competitive, non-competitive, uncompetitive and mixed inhibition available from the GraFit Data Analysis Software v.6.0.12.

### Slow binding inhibition of aldolase A by UM0112176

Residual activity of rabbit muscle aldolase following UM0112176 incubation was determined as follows. UM0112176 (10–100 µM at 25 °C and 5 µM at 37 °C) in 1.0 mL of assay mixture was preequilibrated for 10 min at the appropriate temperature. Aldolase was added to a final concentration of 0.1 mg/mL, and 10 μL aliquots were removed at various times for determination of residual enzyme activity. Control experiments contained no UM0112176. Residual aldolase A activity was measured using 100 µM FBP.

### Inhibition of glycolytic enzymes activity by UM0112176

Enzymes activity was measured in cytosolic extracts prepared from freshly dissected skeletal muscle from adult Swiss white mice. In brief, muscle samples were homogenized with Ultra Turrax T8 homogenizer (IKA Labortechnik) in ice-cold buffer: 20 mM Tris-HCl, 1 mM EDTA, 1 mM EGTA, 1 mM DTT, 60 mM NaF, 1 mM PMFS, 1 g/mL leupeptin, pH 7.4, 4 °C

Enzyme activities were assayed in the extract after 15 min of incubation at 37 °C with UM0112176 using supernatants obtained by centrifugation of the homogenates at 20,000 × *g* at 4 °C for 20 min. Enzyme activity expressed in U [mol min^−1^] was determined from the difference in the slope of NAD(P)H absorbance (340 nm; ε = 6.22 mM^−1^ cm^−1^) before and after addition of a substrate. The activities were measured at 37 °C based on the assays described by Wiśniewski et al.^3^. All enzyme measurements were repeated three times using cells extracts prepared from three independent cell cultures. Statistical significance of differences in the means of control and experiment groups was tested using the T test at significance level of 0.05. Dispersion of measurements was described by standard deviations.

### Western blot

To obtain protein extracts, cells were lysed with 50 mM Tris buffer (pH 8.0) containing 0.2 mM EDTA, 5% SDS and 50 mM DTT for 20 min at 99 °C and centrifuged at 20,000 × g, 20 min, 4 °C. The supernatants were collected, and total protein concentration was determined using the Bradford method. 10 μg of proteins per extract or coimmunoprecipitation reaction were resolved by 10% SDS-PAGE, transferred to a nitrocellulose membrane using wet transfer and stained with Ponceau S to test the quality of the transfer. Membranes were blocked for 1 h with 3% BSA in PBS and then incubated overnight at 4 °C with primary antibodies (rabbit anti-ALDOA, 1:1000, Sigma; rabbit anti NOX-1, 1:3000, Sigma) diluted in PBS. The membranes were then incubated for 1 h in RT with secondary antibodies (goat anti-rabbit IgG-HC, HRP conjugated, 1:1000, Sigma) diluted in PBS. Rabbit anti-β-actin (1:3,000, Sigma) and IgG heavy chains were used as a loading control in experiments with, respectively, cellular extracts and coimmunoprecipitation. A peroxidase substrate, 3,3’-diaminobenzidine (DAB), was used to develop a color reaction.

### Coimmunoprecipitation

9.5 µg of recombinant human cofilin (Cytoskeleton Inc.), 19 µg of recombinant human aldolase A (approx. 1:1 molar ratio) and either 10 µM UM0112176 or DMSO were incubated overnight in PBS in 4 °C with gentle mixing. Next, the mixtures were incubated with 5 µg of rabbit anti-cofilin antibodies (Sigma) for 8 h in 4 °C. Finally, the mixtures were incubated with 50 µL of protein G agarose beads (Roche) overnight in 4 °C. Protein complexes bound to protein G agarose were precipitated using centrifugation at 12,000 × g, 1 min; suspended in 50 µL SDS-PAGE loading buffer, denatured in 99 °C for 10 min and analyzed using western blot with primary antibodies specific to aldolase.

### Aldolase-actin binding

83 µg of platelet-derived human β/γ-actin (Cytoskeleton Inc.) per sample was polymerized according to the manufacturer’s protocol in 15 mM Tris-HCl (pH 7.5) with 50 mM KCl, 2 mM MgCl_2_, 0.2 mM CaCl_2_, 0.5 mM DTT and 1.2 mM ATP. 50 µg of human recombinant ALDOA was preincubated with either 10 µM UM0112176 or DMSO (15 min) and then added to actin (total volume of 250 µl per sample). Samples containing only actin or aldolase were used as additional control. All samples were then incubated for 15 min in RT. F-actin was separated from the solution by ultracentrifugation at 100,000 × *g*, 1 h, 4 °C. The pellet was resuspended in a volume of actin polymerization buffer equal to the volume of the supernatant. Enzymatic activity of ALDOA was measured in pellets and supernatants of all samples as described in the “Kinetics of aldolase A in the presence of UM0112176” section.

### Actin depolymerization assay

The effect of UM0112176 and human muscle cofilin 1 (Cytoskeleton Inc.) on actin depolymerization was studied through the rate of fluorescence decrease that occurs during pyrene-conjugated F-actin conversion into G-actin using Actin Polymerization Biochem Kit™ (Cytoskeleton Inc.).

Rabbit β/γ pyrene-conjugated F-actin was prepared according to the manufacturer’s manual and the effect of various molecules on the depolymerization was monitored using fluorescence spectrophotometer (Varioskan™ LUX multimode microplate reader).

The final concentration of UM0112176 was 10 µM while aldolase A and cofilin were 1 µM. Monomer concentration of β/γ pyrene-conjugated F-actin was 4.7 µM.

### Molecular docking

Complex formation between UM0112176 and ALDOA was investigated with Auto Dock v4.2^32^. The protein target was processed by adding all hydrogens and merging non-polar hydrogen atoms using AutoDock Tools. The charges were assigned using the Gasteiger method and a semiflexible docking protocol was followed. The protein molecule was kept rigid, and ligand torsions were treated as flexible about rotatable bonds. After pre-calculation of the grid with autogrid, autodock was performed using a Lamarckian algorithm. The pose with best binding affinity was visualized using PyMol (The PyMOL Molecular Graphics System, Version 2.0 Schrödinger, LLC).

### Statistical analysis

#### Immunocytochemistry and measurement of cellular ROS and Ca^2+^ content

Details of statistical analysis are provided in the respective subsections of Methods. Significance level and number of samples are provided in the appropriate figure legends.

#### MTT assay

In all experimental conditions, measurements were averaged from at least eight wells. Statistical significance of differences in the means of control and experiment groups was tested using the T test at significance level of 0.05. Dispersion was described using standard deviation. The experiments were performed in triplicate, with similar results.

#### Fructose-1,6-bisphosphate and triose phosphates concentration in astrocytes and KLN205 cells

All measurements were repeated three times using cells extracts prepared from three independent cell cultures. Statistical significance of differences in the means of control and experiment groups was tested using the T test at significance level of 0.05. Measurement dispersion was described by standard deviations.

#### Aldolase-actin binding

All measurements were repeated three times. Statistical significance of differences in the means of control and experiment groups was tested using the T test at significance level of 0.05. Measurement dispersion was described by standard deviations.

#### Kinetics of aldolase A in the presence of UM0112176

The inhibition kinetics was then analyzed using the enzyme models for competitive, non-competitive, uncompetitive and mixed inhibition available from the GraFit Data Analysis Software v.6.0.12. The mixed inhibition model yielded the best fit to the inhibition data based on the chi-square residual. The corresponding reciprocal plots were linear within experimental error with greatest deviation occurring at lowest FBP concentration used to assess residual activity (5 μM).

## Supplementary information

Figure S1

Figure S2

Figure S3

Figure S4

Figure S5

Supplementary figure legend
