# Supplementary material for: Targeting a moonlighting function of aldolase induces apoptosis in cancer cells
Source: Cell Death Dis. 2019 Sep 26;10(10):712. doi: 10.1038/s41419-019-1968-4 (PMC6763475; doi:10.1038/s41419-019-1968-4)
Supplement: Supplementary file 1 — Figure S1 [file 41419_2019_1968_MOESM1_ESM.pdf]

a)

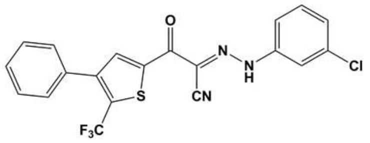

(*E*)-*N*'-(3-chlorophenyl)-2-oxo-2-(4-phenyl-5-(trifluoromethyl)thiophen-2-yl)acetohydrazonoyl cyanide
Chemical Formula: $C_{20}H_{11}ClF_3N_3OS$
Molecular Weight: 433.83
avg LogP 6.58 (1.35)
avg LogS -6.48 (~ 0.2mg/L)

ClC1=CC(N/N=C(C#N)/C(C2=CC(C3=CC=CC=C3)=C(C(F)(F)F)S2)=O)=CC=C1

b)

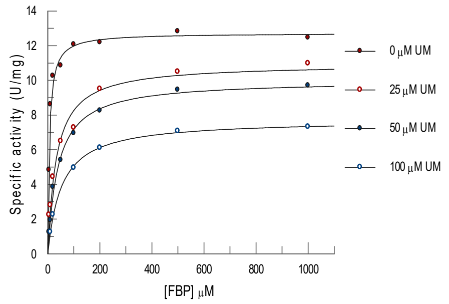

$$v = \frac{V_{max} \cdot [S]}{K_m\left(1+\frac{[I]}{K_i}\right)+\left(1+\frac{[I]}{K_i'}\right)[S]}$$

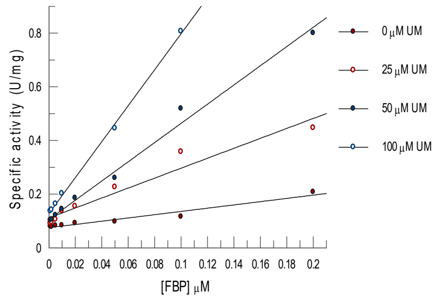

Equation:	Mixed Inhibition
Weighting:	Simple
Data points:	30
Chi²:	3.4919
Reduced Chi²:	0.1343

Parameter	Value	Std. Error
Vmax	12.6740	0.1813
Km	6.0021	0.6127
Ki	6.2195	0.8114
Ki' value	175.0644	17.0535

c)

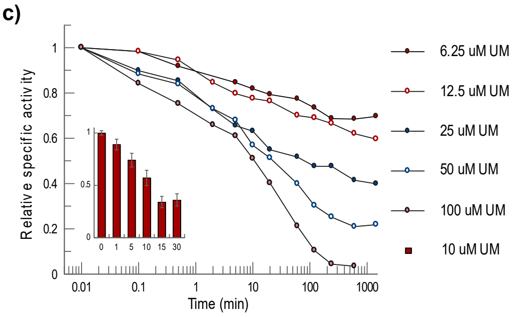

d)

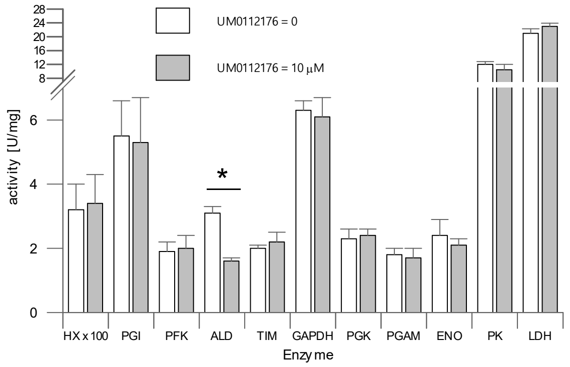

e)

	FBP		P-TRIOSES	
	µM	SD	µM	SD
**KLN205**				
Full medium	60*	22	153*	46
Full medium+UM0112176	199	38	78	20
glutamine only	50*	11	99*	28
glutamine+UM0112176	34	10	223	29
glucose only	64*	23	100*	11
glucose+UM0112176	137	25	89	10
**ASTROCYTES**				
Full medium	617**	91	933*	224
Full medium+UM0112176	1155	109	279	62
glutamine only	292*	49	1038*	99
glutamine+UM0112176	250	46	3158	566
glucose only	476*	61	565*	113
glucose+UM0112176	847	140	347	97

n=3 (three independent cells culturing experiments)
* - p<0.05
** - p<0.005
1 - full medium (glucose – 5 mM, glutamine- 2 mM)
2 - full medium+UM0112176
3 – glutamine (2 mM) only
4 – glutamine only+UM0112176
5 – glucose (5 mM) only
6 – glucose only + UM0112176
