# Supplementary material for: Targeting a moonlighting function of aldolase induces apoptosis in cancer cells
Source: Cell Death Dis. 2019 Sep 26;10(10):712. doi: 10.1038/s41419-019-1968-4 (PMC6763475; doi:10.1038/s41419-019-1968-4)
Supplement: Supplementary file 2 — Figure S2 [file 41419_2019_1968_MOESM2_ESM.pdf]

a)

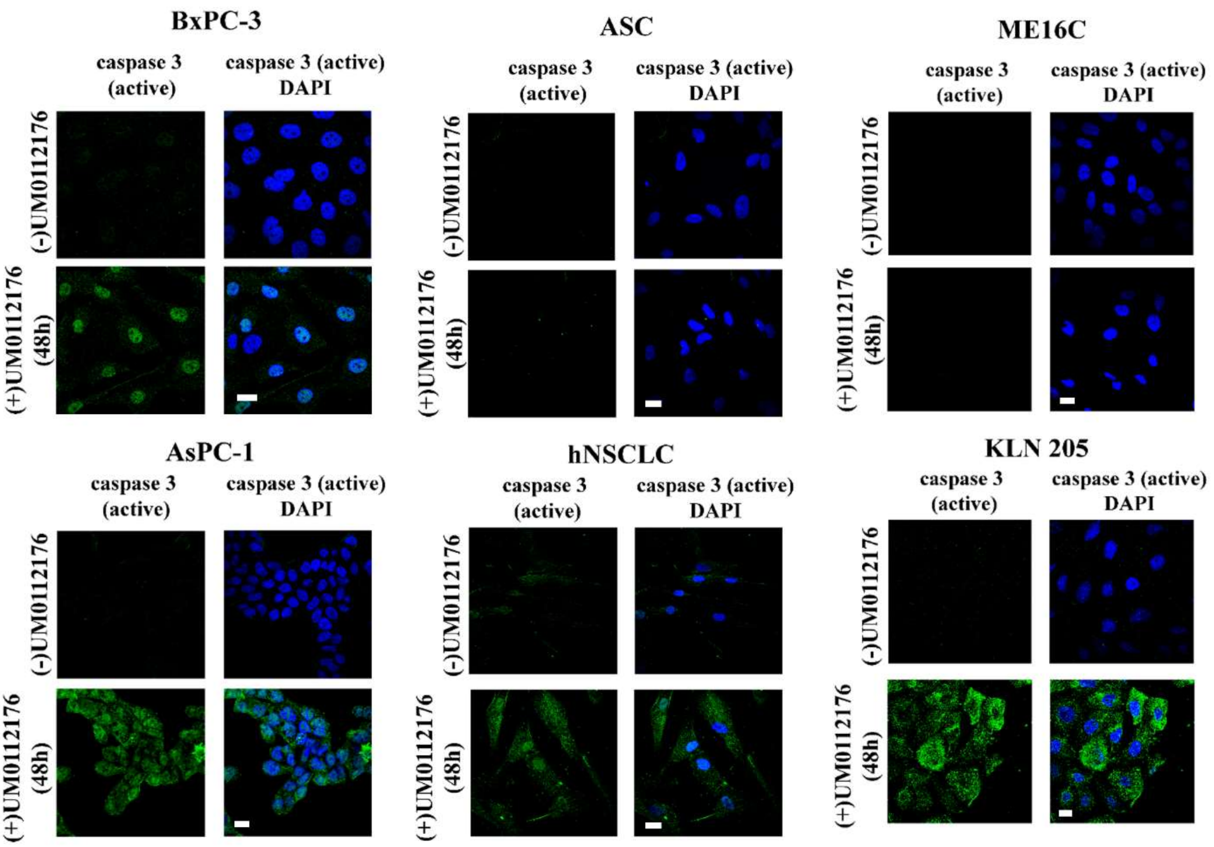

BxPC-3 - human primary pancreatic adenocarcinoma; ASC – primary culture of rat astrocytes; Me16C - epithelial cells immortalized with hTERT; AsPC-1 – from human pancreas adenocarcinoma ascites metastasis; KLN205 – mouse lung squamous cell carcinoma; hNSCLC – human small cell lung squamous cancer

b)

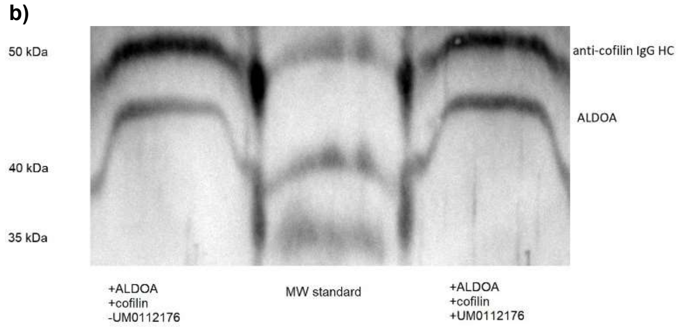

c)

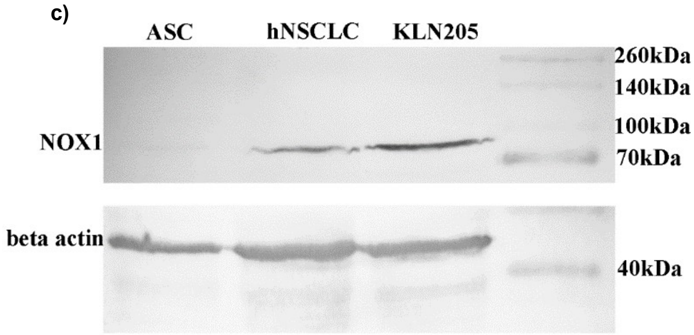

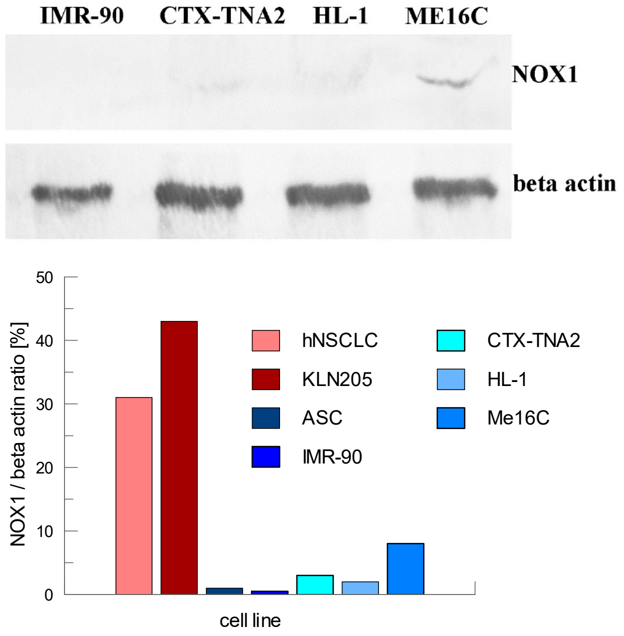

ASC – primary culture of rat astrocytes

hNSCLC – human small cell lung squamous cancer

KLN205 – mouse squamous cell carcinoma

CTX-TNA2 - rat astrocyte, transfected

IMR-90 - human embryonic lung fibroblasts

HL-1 – mouse cardiomyocytes

ME16C - epithelial cells immortalized with hTERT
