# Supplementary figures and images for: Targeting a moonlighting function of aldolase induces apoptosis in cancer cells
Source: Cell Death Dis. 2019 Sep 26;10(10):712. doi: 10.1038/s41419-019-1968-4 (PMC6763475; doi:10.1038/s41419-019-1968-4)

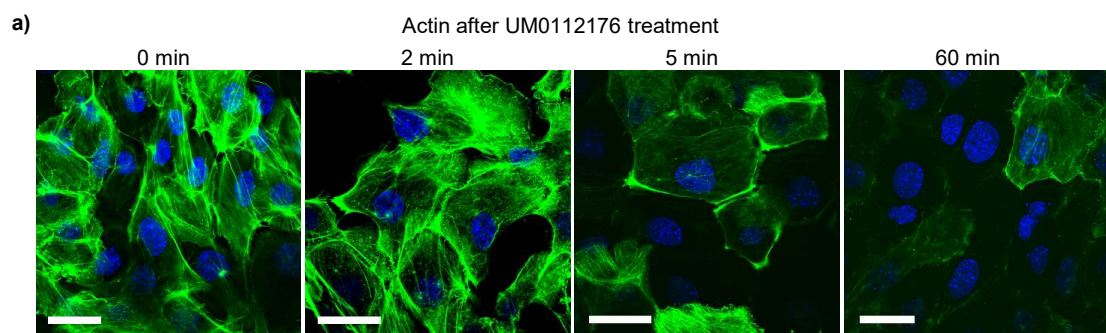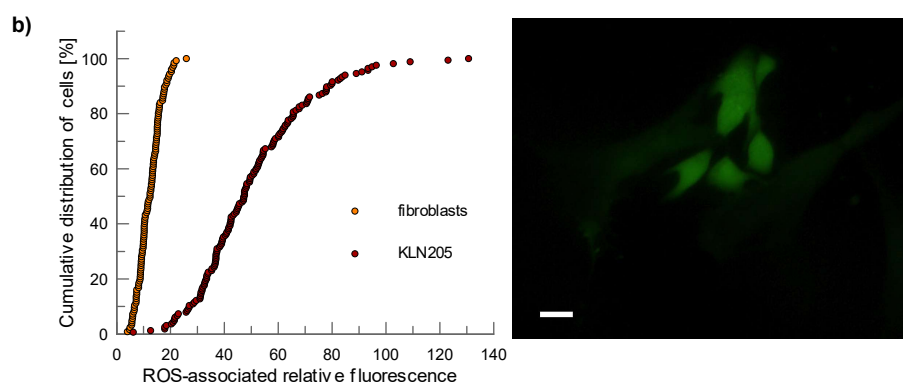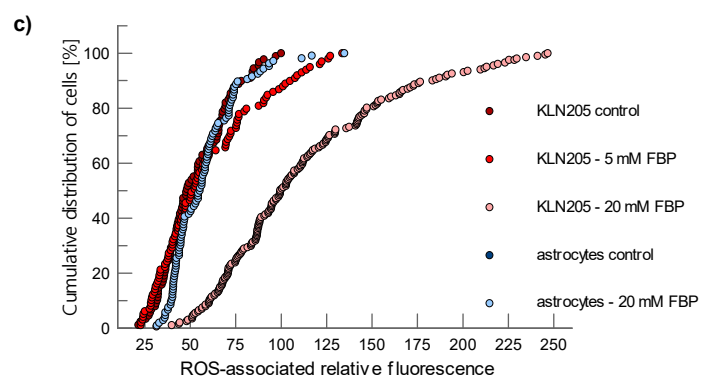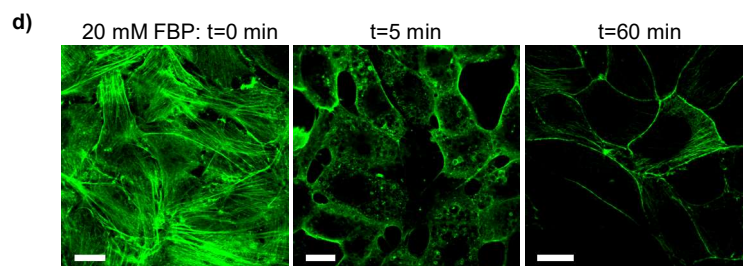

Supplement: Supplementary file 3 — Figure S3 [file 41419_2019_1968_MOESM3_ESM.pdf]

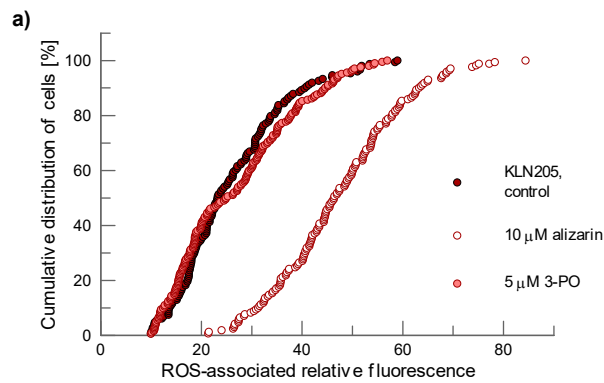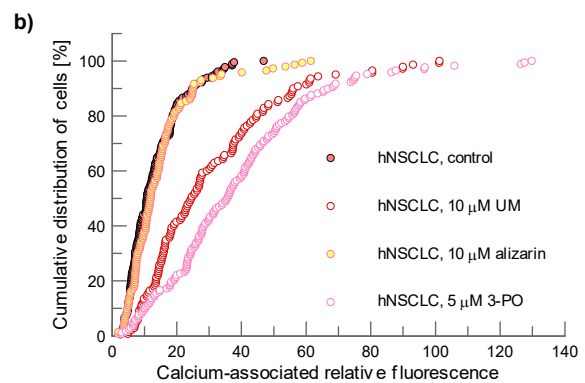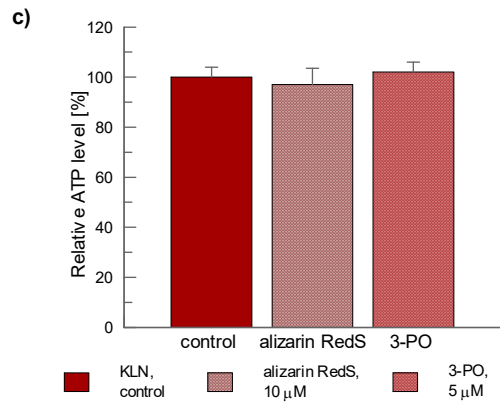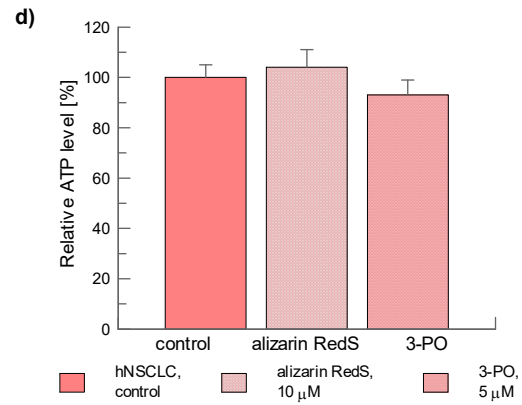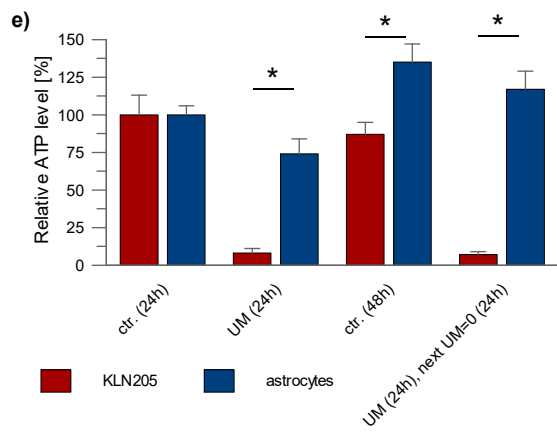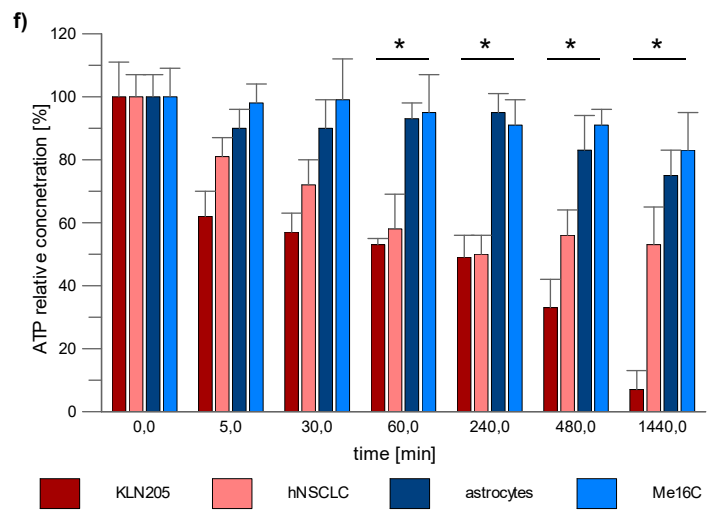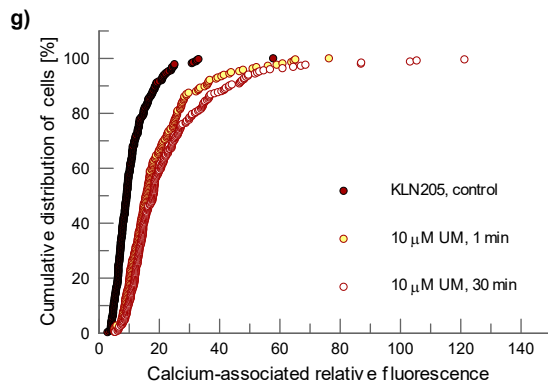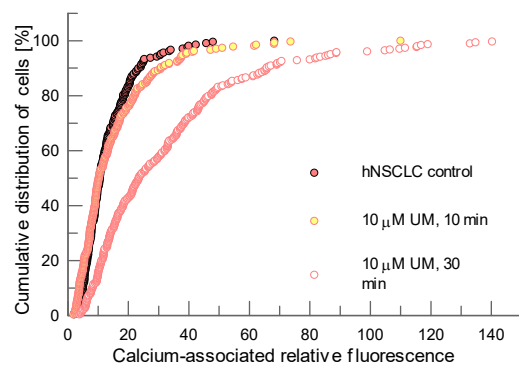

Supplement: Supplementary file 4 — Figure S4 [file 41419_2019_1968_MOESM4_ESM.pdf]

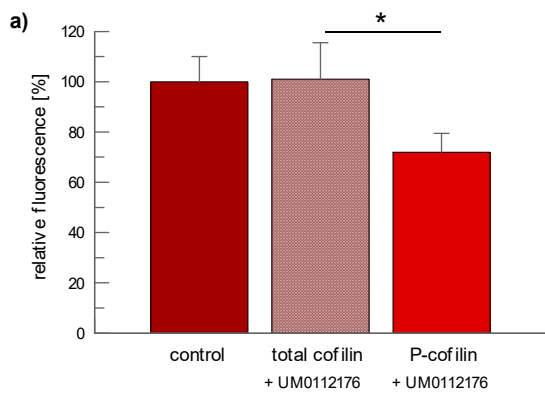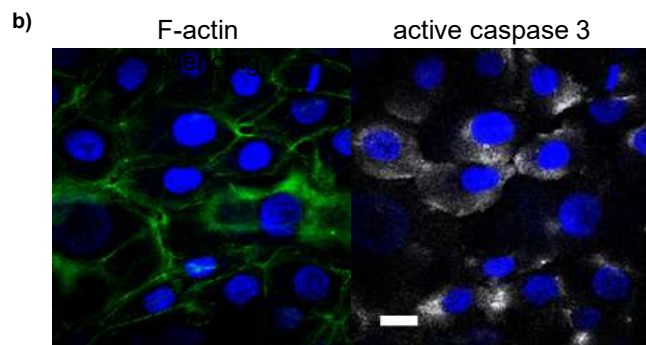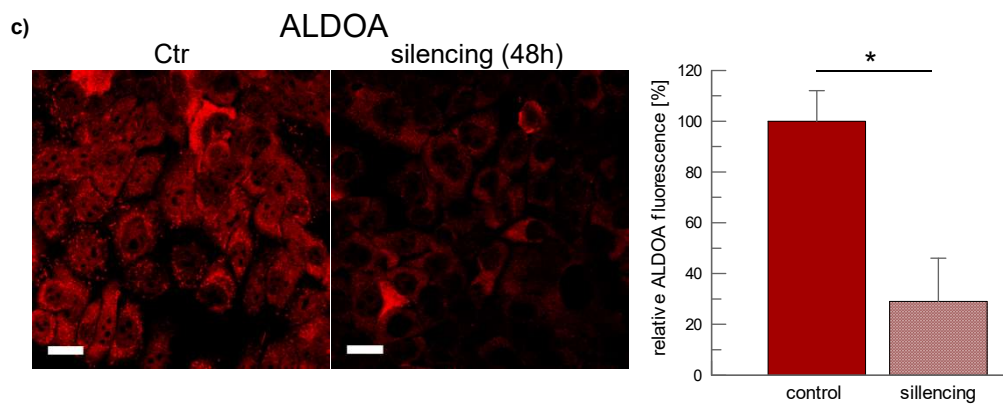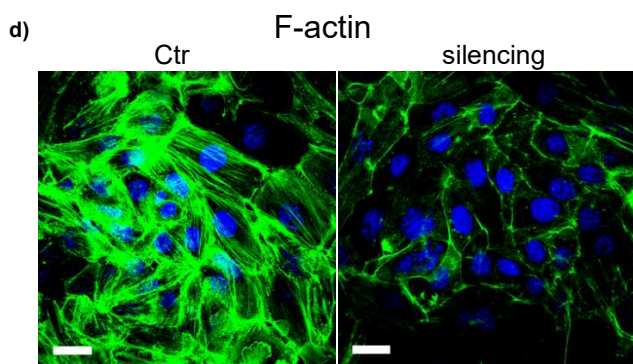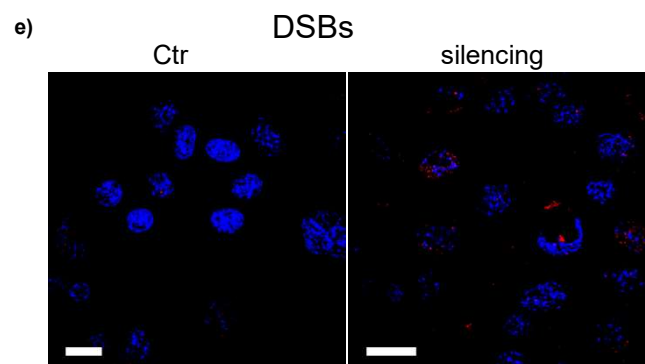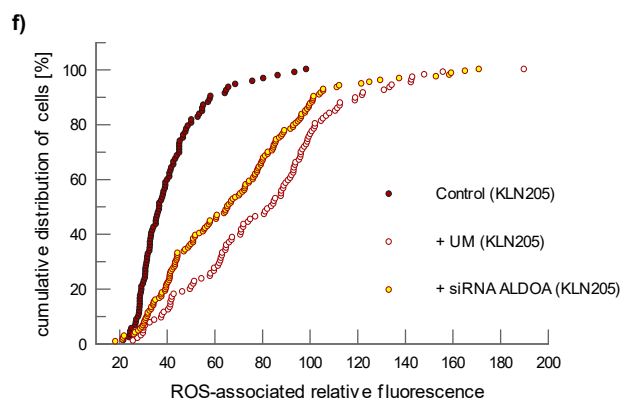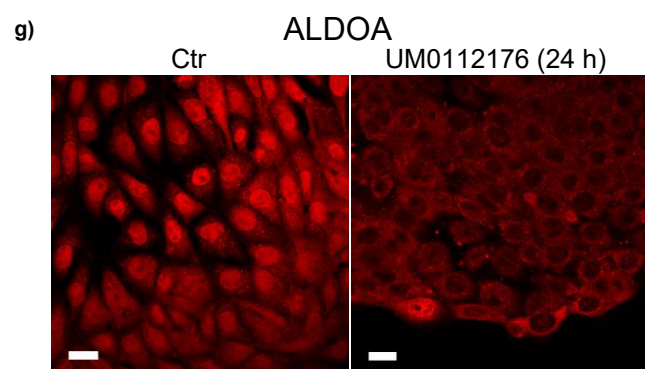

Supplement: Supplementary file 5 — Figure S5 [file 41419_2019_1968_MOESM5_ESM.pdf]
