# Supplementary material for: Targeting a moonlighting function of aldolase induces apoptosis in cancer cells
Source: Cell Death Dis. 2019 Sep 26;10(10):712. doi: 10.1038/s41419-019-1968-4 (PMC6763475; doi:10.1038/s41419-019-1968-4)
Supplement: Supplementary file 6 — Supplementary figure legend [file 41419_2019_1968_MOESM6_ESM.docx]

**Supplementary Figure Legends**

**Figure S1**

**UM0112176 inhibition kinetics**

a) Chemical structure of the slow-binding inhibitor, UM0112176.

b) Mixed inhibition kinetics. Aldolase A activity was determined in the presence of UM0112176 and immediately assaying residual activity at various concentrations of substrate, fructose-1,6-bisphosphate (FBP).

c) Slow binding inhibition of Aldolase A by UM0112176. Relative aldolase A activity was measured at 25°C using 100 µM FBP following incubation with UM0112176. Inset: Relative aldolase A activity measured at 37°C using 100 µM FBP as a substrate following incubation with UM0112176.

d) Inhibition of glycolytic enzymes by UM0112176. The values are given as a mean from 3 independent measurements and SD, * – P<0.05.

e) The effect of 10 μM UM0112176 (24 h of treatment) on FBP and triose phosphates (P-Trioses) concentration in extracts from KLN205 and primary culture of rat astrocytes.

**Figure S2**

**Understanding UM0112176-induced changes in cancer cells**

a) The effect of 10 μM UM0112176 (48 h of treatment) on caspase 3 activation in cancer and normal cells. Bar=10 µm.

b) ALDOA-cofilin interaction in vitro studied using co-immunoprecipitation.

c) NOX1 expression in different cells.

**Figure S3**

**Effect of UM0112176 and FBP treatment on the cancer cell actin cytoskeleton and on ROS levels**

a) Images of actin cytoskeleton in KLN205 cells after UM0112176 treatment taken at t= 0, 2, 5 and 60 min). Bar=20 µm.

b) ROS generated in co-culture of fibroblasts and KLN205 after UM0112176 treatment. The image (right) shows only cell fluorescence associated with KLN205 cells. Bar=20 µm.

c) FBP-stimulated ROS production in cancer and normal cells.

d) FBP-destabilized cytoskeleton in KLN205 cancer cells. Bar=20 µm.

**Figure S4**

**The effects of glycolysis inhibition on ATP and Ca2+ levels in normal and cancer cells** Values shown in bar graphs have been normalized either to the untreated control or to t=0

a) The effect of 3-PO and alizarin RedS on ROS levels in KLN205.

b) The effect of 3-PO and alizarin RedS on Ca2+ level in hNSCLC.

c) The effect of 3-PO and alizarin RedS on ATP levels in KLN205.

d) The effect of 3-PO and alizarin RedS on ATP levels in hNSCLC.

e) The reversibility of UM0112176-induced decrease in ATP levels in normal but not in cancer cells after withdrawal of the inhibitor. The values are given as a mean from 3 independent experiments and SD, * – P<0.05.

f) The changes in ATP levels after UM0112176 treatment in cancer (KLN205 and hNSCLC) and normal cells (astrocytes and immortalized epithelial cells). The values are given as a mean from 3 independent experiments and SD, * – P<0.05 for all normal cell lines vs all cancer cell lines.

g) Time-dependence of changes in calcium levels in KLN205 and hNSCLC cells.

**Figure S5**

**UM0112176 treatment on P-cofilin levels and NCX activity; cellular effects of ALDOA silencing in KLN205 cells**

a) The levels of total cofilin and P-cofilin before and after UM0112176 treatment. The values are given as a mean and SD, * – P<0.05.

b) Inhibition of NCX does not block UM0112176-induced caspase activation (white) and cytoskeletal actin disruption (green). Bar=10 µm.

c) ALDOA silencing in the KLN205 – the level of the enzyme treated with antisense shRNA – a control (left) and after 48 h of the silencing with specific shRNA against ALDOA (right). Bar=10 µm. The graph shows a decrease in ALDOA-associated fluorescence after silencing.

d) Silencing of ALDOA disrupts actin cytoskeleton (left – control, right after 48 h of the silencing). Bar=10 µm.

e) DSBs generation in the control cells (left) and in cells with silenced expression of ALDOA. Bar=10 µm.

f) The effect of ALDOA expression silencing and UM0112176 treatment on ROS generation.

g) 10 µM UM0112176 treatment (24 h) stimulates ALDOA withdrawal from cancer cells’ nuclei. Bar=10 µm.
